# Perturbed development of cranial neural crest cells in association with reduced sonic hedgehog signaling underlies the pathogenesis of retinoic-acid-induced cleft palate

**DOI:** 10.1242/dmm.040279

**Published:** 2019-10-01

**Authors:** Qi Wang, Hiroshi Kurosaka, Masataka Kikuchi, Akihiro Nakaya, Paul A. Trainor, Takashi Yamashiro

**Affiliations:** 1 Department of Orthodontics and Dentofacial Orthopedics, Graduate School of Dentistry, Osaka University, Suita 565-0871, Japan; 2 Department of Genome Informatics, Graduate School of Medicine, Osaka University, Suita 565-0871, Japan; 3 Stowers Institute for Medical Research, Kansas City, MO 64110, USA; 4 Department of Anatomy and Cell Biology, University of Kansas Medical Center, Kansas City, KS 66160, USA

**Keywords:** Retinoic acid, Shh, Vitamin A, Embryogenesis, Apoptosis, Craniofacial development

## Abstract

Cleft palate (CP) is one of the most common congenital craniofacial anomalies in humans and can be caused by either single or multiple genetic and environmental factor(s). With respect to environmental factors, excessive intake of vitamin A during early pregnancy is associated with increased incidence of CP in offspring both in humans and in animal models. Vitamin A is metabolized to retinoic acid (RA); however, the pathogenetic mechanism of CP caused by altered RA signaling during early embryogenesis is not fully understood. To investigate the detailed cellular and molecular mechanism of RA-induced CP, we administered all-trans RA to pregnant mice at embryonic day (E)8.5. In the RA-treated group, we observed altered expression of *Sox10*, which marks cranial neural crest cells (CNCCs). Disruption of *Sox10* expression was also observed at E10.5 in the maxillary component of the first branchial arch, which gives rise to secondary palatal shelves. Moreover, we found significant elevation of CNCC apoptosis in RA-treated embryos. RNA-sequencing comparisons of RA-treated embryos compared to controls revealed alterations in Sonic hedgehog (Shh) signaling. More specifically, the expression of *Shh* and its downstream genes *Ptch1* and *Gli1* was spatiotemporally downregulated in the developing face of RA-treated embryos. Consistent with these findings, the incidence of CP in association with excessive RA signaling was reduced by administration of the Shh signaling agonist SAG (Smoothened agonist). Altogether, our results uncovered a novel mechanistic association between RA-induced CP with decreased Shh signaling and elevated CNCC apoptosis.

## Abstract (precis)

**Editor's choice:** The RA-Shh signaling pathway is revealed to be critical during cranial neural crest development, which impacts secondary palate development.

## INTRODUCTION

Isolated cleft palate (CP) is one of the most common birth defects in humans, and occurs with an incidence of 1 in 1000 live births (Cox, 2004). The underlying causes of CP are multifactorial, and involve various genetic and environmental factors (Dixon et al., 2011). For example, retinoic acid (RA) signaling is well known to play critical roles during craniofacial development, with both gain and loss of function resulting in CP both in humans and animal models (Abbott et al., 1989; Ackermans et al., 2011; Lammer et al., 1985).

Classic animal experiments revealed that excessive vitamin A and RA signaling during palatogenesis results in CP. Interestingly, this effect depends on the time of RA exposure. RA treatment on embryonic day (E)10 produces small palatal shelves that fail to contact each other, whereas treatment on E12 results in normal-sized palatal shelves that contact each other, but do not fuse due to disturbance of the epithelium (Abbott et al., 1989). However, relatively few studies have investigated the effect of RA signaling at earlier developmental stages (younger than E10) on the etiology and pathogenesis of CP. In the present study, we discovered that excessive RA signaling at E8.5 of mouse embryogenesis results in a higher incidence of CP, compared to embryos subjected to RA treatment at E10 or later. The higher relative incidence of CP was associated with significant cranial neural crest cell (CNCC) apoptosis.

CNCCs are a migratory stem and progenitor cell population which arises from the dorsolateral neuroepithelium during neurulation and contributes to the majority of the cartilage, bone, connective tissue and peripheral nerve tissue in the head. CNCC development involves a sequence of critical phases, such as formation, migration and differentiation. Problems in one or more of these events can result in craniofacial abnormalities. For example, in Treacher Collins syndrome, neuroepithelial apoptosis and decreased neural crest cell (NCC) proliferation results in a reduction in the number of migrating CNCCs, which culminates in cranioskeletal hypoplasia and CP (Conley et al., 2016; Dixon et al., 2006; Jones et al., 2008; Sakai et al., 2016).

In the present study, we observed a substantial reduction of Sonic hedgehog (Shh) signaling during craniofacial development in embryos treated with exogenous RA. Shh protein is an important secreted signaling molecule known to function in the patterning of a variety of cells and tissues during embryonic development. Shh signaling is activated in target cells through binding of Shh ligand to the transmembrane receptor Patched1 (Ptch1), which de-represses the activity of Smoothened (Smo) (Casali and Struhl, 2004). Notably, *Shh* is expressed in the pre-chordal plate, the notochord and the floor plate of the neural tube during neurulation, which encompasses the period of NCC formation and migration. Furthermore, inhibiting Shh signaling during chick embryo development has been shown to cause reduced head size, hypoplasia or agenesis of the pharyngeal arches, and these phenotypes are associated with elevated cell death in neural tube and NCCs (Ahlgren and Bronner-Fraser, 1999).

In this study, we show that, during mouse embryonic development, the E8.5 developmental stage is very sensitive to maternal RA-exposure-induced CP. This phenotype is associated with diminished survival of CNCCs, which led to defects in first pharyngeal arch development. RNA-sequencing (RNAseq) analyses revealed that this phenotype was associated with reduced Shh signaling. Taken together, our results have revealed novel molecular and cellular mechanisms linking elevated RA with decreased Shh signaling, which compromises the survival of CNCCs and causes CP. Consistent with these observations, restoration of Shh signaling via a chemical agonist diminishes CNCC apoptosis and reduces the incidence of CP.

## RESULTS

### Excessive maternal RA intake induces cleft palate with different probability according to embryonic stage

**Fig. 1.**
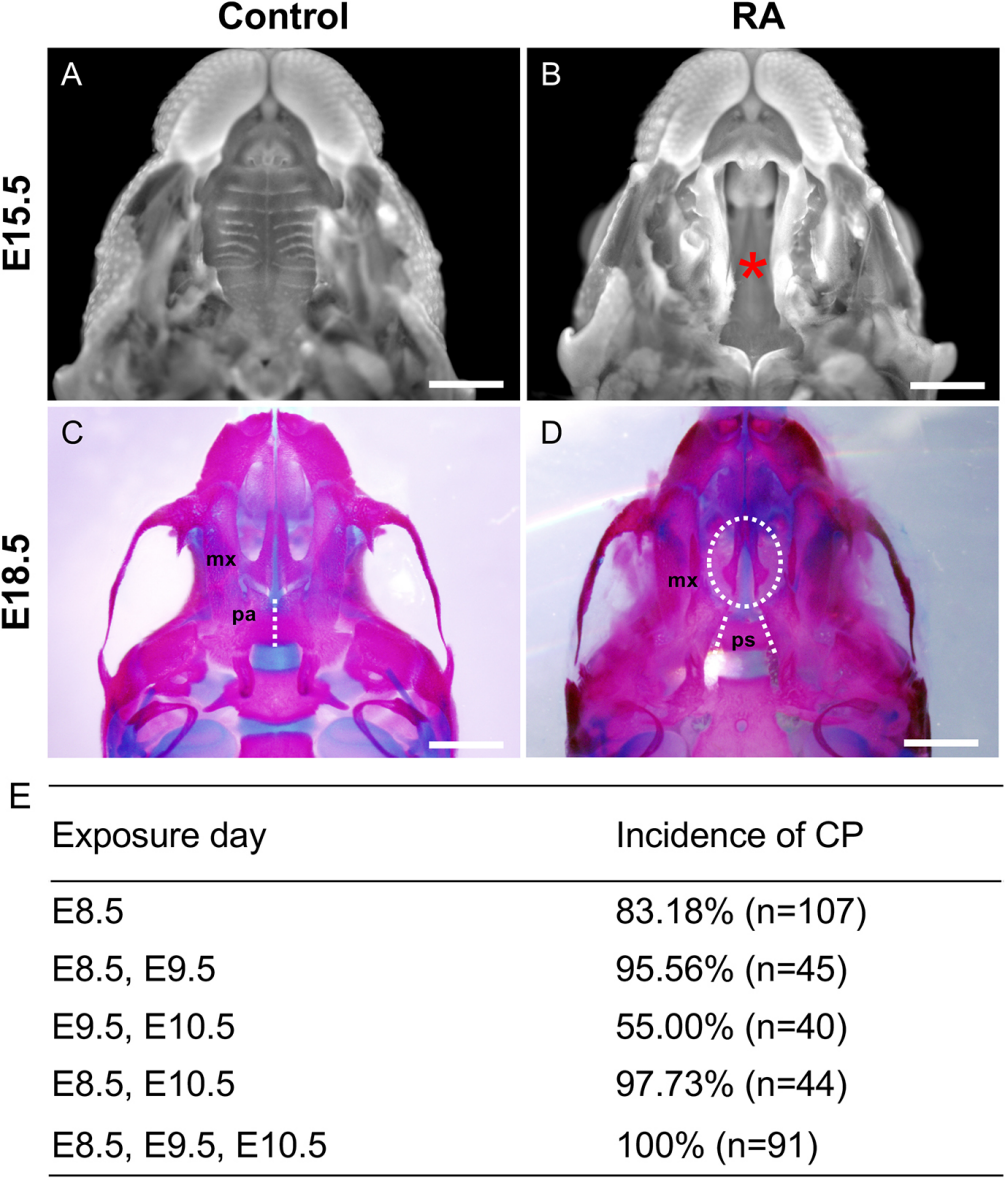
**Cleft palate (CP) in RA-treated embryos.** (A,B) DAPI-stained upper jaws of control (A) and RA-treated (B) embryos at E15.5 are shown. The asterisk in B marks the CP in RA-treated embryos. (C,D) Skeletal preparations of E18.5 control (C) and RA-treated (D) embryos stained with Alizarin Red for mineralized bone and Alcian Blue for cartilage. The palatal shelves in the maxilla (mx) of RA-treated embryos with complete CP (oval dashed line in D) failed to grow toward the midline. Fusion of the bilateral palatal bones (pa) is observed in the control embryos (dashed line in C) but the presphenoid bone (ps) of the cranial base is fully exposed in the RA-treated embryo skeleton due to CP (dashed lines in D). (E) The incidence of CP with all-trans RA (25 mg/kg maternal body weight) treatment at different embryonic days. *n*, number of embryos analyzed; %, percentage of embryos analyzed. Scale bars: 1 mm.

Although RA-induced CP has previously been investigated in an embryonic-stage-dependent manner, the administration of RA was performed mainly after E10 (Abbott et al., 1989; Pennimpede et al., 2010). Thus, our knowledge is still meager in terms of the mechanism of early-stage RA-induced CP. In the present study we orally gavaged pregnant mice with RA (25 mg/kg body weight) at different gestational time points to investigate the embryonic-stage-dependent effect of excessive RA signaling on secondary palate development (from E8.5 to E10.5) (Fig. 1E). Compared to untreated control embryos, all of which displayed normal palate development, many of the embryos from mothers exposed to exogenous RA at any time point from E8.5 to E10.5 exhibited CP. More specifically, the embryos exposed to RA at E8.5 exhibited a higher rate of CP than embryos treated at other time points (Fig. 1E). Based on these data, we hypothesized that excess RA signaling at E8.5 perturbs critical cellular events, leading to CP. Thus, we chose RA exposure (25 mg/kg maternal weight) at E8.5 for further investigation of CP in an attempt to understand the embryological basis of this anomaly.

Dissection of RA-treated embryos at E15.5 revealed the presence of a complete cleft of the secondary palate (Fig. 1B). Skeletal staining demonstrated that both the maxillary and palatine shelves exhibited a complete cleft (Fig. 1D). As a result, the presphenoid bone was visible in the ventral view (Fig. 1D), while the palatal bones were fused with each other and the maxillary processes located at the midline in the control embryos (Fig. 1C).

### Impaired CNCC development contributes to RA-induced cleft palate

**Fig. 2.**
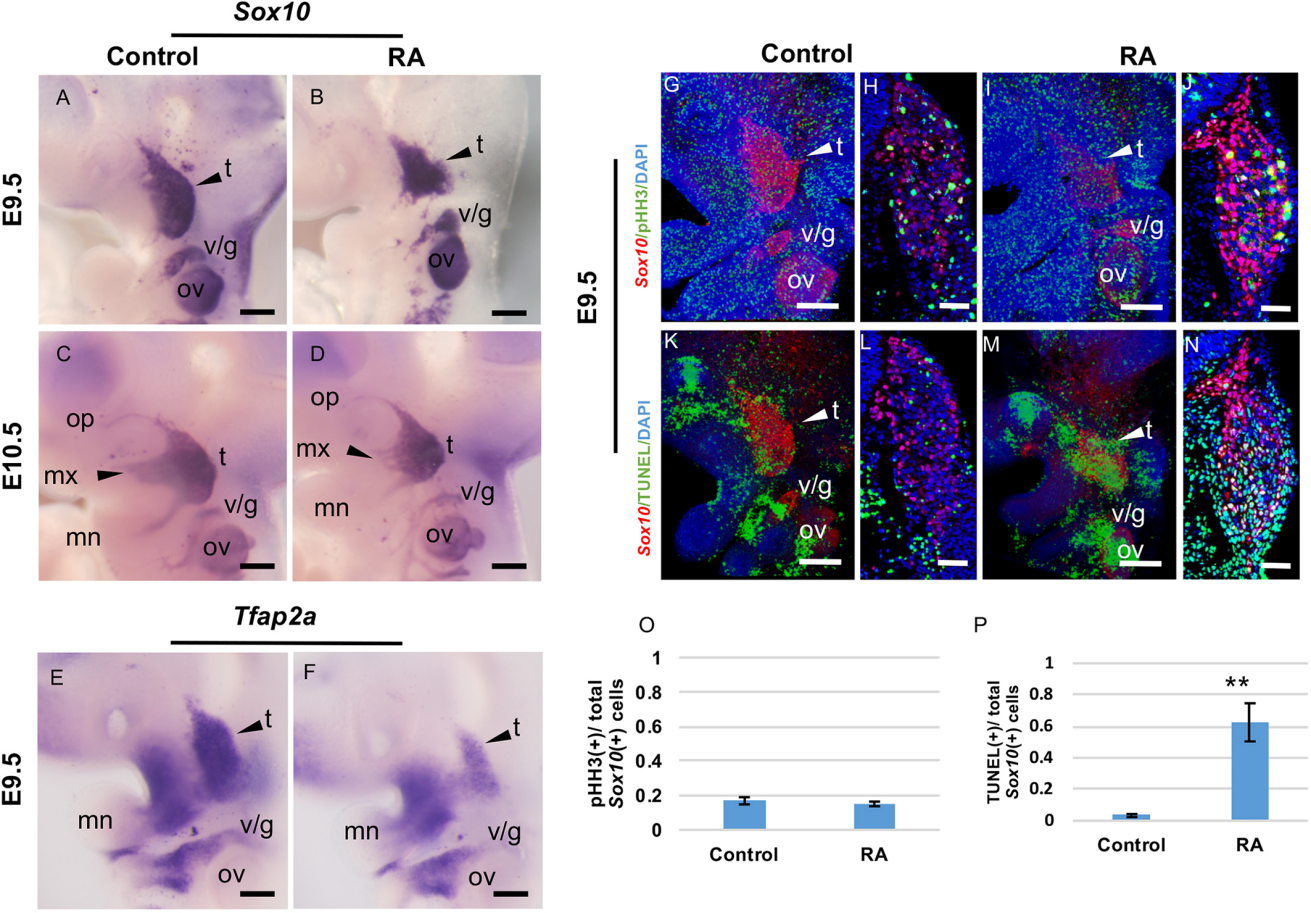
**Excess RA leads to defects of *Sox10*-positive CNCCs.** (A-D) Whole-mount *in situ* hybridization for *Sox10* (A-D) and *Tfap2a* (E,F) at E9.5 (A,B,E,F) and E10.5 (C,D) in control (A,C,E) and RA-treated (B,D,F) embryos. (A,B) The arrowheads represent the *Sox10* expression domain in the trigeminal ganglia at E9.5. The *Sox10* staining pattern in RA-treated embryos (B) is smaller. (C,D) The arrowheads show CNCC migration into the maxillary component in the first branchial arch. The stream of RA-treated embryos (D) is disturbed. (E,F) *Tfap2a* expression was decreased in the maxillary region of RA-treated embryos. (G-P) Unaltered proliferation but increased apoptosis in *Sox10*-positive (red) trigeminal ganglia (arrowheads) at E9.5 after RA treatment. Whole-mount immunofluorescent detection of pHH3 (green; G,I) and TUNEL (green; K,M) in profile views of control (G,K) and RA-treated (I,M) E9.5 embryos. Immunostaining of pHH3 (green; H,J) and TUNEL (green; L,N) of transverse sections of control (H,L) and RA-treated (J,N) E9.5 embryos at the level of the trigeminal ganglia. (O,P) Quantification of proliferation (pHH3) and apoptosis (TUNEL) in *Sox10*-positive CNCCs from control and RA-treated embryos in the trigeminal ganglia regions. *t*-test **P<0.01, *n*=5. Data are mean±s.e.m. *n*=3 for each stage and experiment. t, trigeminal ganglion; v/g, vestibulo-cochlear/geniculate ganglia; ov, otic vesicle; op, ophthalmic branch; mn, mandibular branch; mx, maxillary branch. Scale bars: 100 µm (A-G, I, K and M); 50 µm (H, J, L and N).

CNCCs give rise to the majority of the skeletal elements of the head, including the palatal bones (Santagati and Rijli, 2003). Interestingly, the E8.5 stage, at which time the embryos were exposed to exogenous RA, coincides with the formation and onset of migration of CNCCs (Trainor, 2005). We therefore hypothesized that RA-induced CP may result from defects in CNCC development. To test this hypothesis, we initially performed *in situ* hybridization using a probe for *Sox10*, which delineates migrating CNCCs at E8.5 and then persists through E9.5 in those cells fated for neurological differentiation (Marmigere and Ernfors, 2007). In an E9.5 control embryo, *Sox10* expression is confined to the cranial ganglia, which project their nerves into the branchial arches between E9.5 and 10.5 (Fig. 2A,C). In contrast, RA-treated embryos exhibited perturbed *Sox10* expression. The trigeminal ganglia (Fig. 2B, arrowhead) are hypoplastic at E9.5, with reduced maxillary extensions into the anterior region of the maxillary component of the first branchial arch at E10.5 (Fig. 2D, arrowhead). We also detected decreased expression of *Tfap2a*, another marker for CNCCs, in RA-treated embryos at E9.5 (Fig. 2E,F). These results indicate that excessive RA signaling at E8.5 disturbs proper CNCC and cranial ganglia development.

To better understand how CNCC development is affected by exposure to exogenous RA, we performed immunohistochemistry with antibodies against Sox10 and phosphorylated histone H3 (pHH3) or TUNEL staining to assess any alterations in proliferation or apoptosis in the CNCC population. Similar to our findings obtained by *in situ* hybridization, a substantial reduction in the domain of Sox10-labeled CNCCs could be observed at E9.5 (Fig. 2G-N). Interestingly, the number of dividing CNCCs in control and RA-treated embryos was essentially the same (Fig. 2G-J,O). However, we observed a notable elevation of cell death in RA-treated embryos compared to controls (Fig. 2K,M, arrowhead). Elevated cell death was confirmed by quantitative analysis of TUNEL staining in conjunction with Sox10 immunolabeling of CNCCs in transverse sections (Fig. 2L,N,P; *P*=0.008). These results strongly suggest that exposure to excessive RA at E8.5 results in CNCC apoptosis, which could contribute to a wide variety of craniofacial defects, including a high incidence of CP. We also detected smaller maxillary processes of the first arch in RA-treated embryos (Fig. 2A-N).

### RA exposure affects Shh signaling in the craniofacial region

**Fig. 3.**
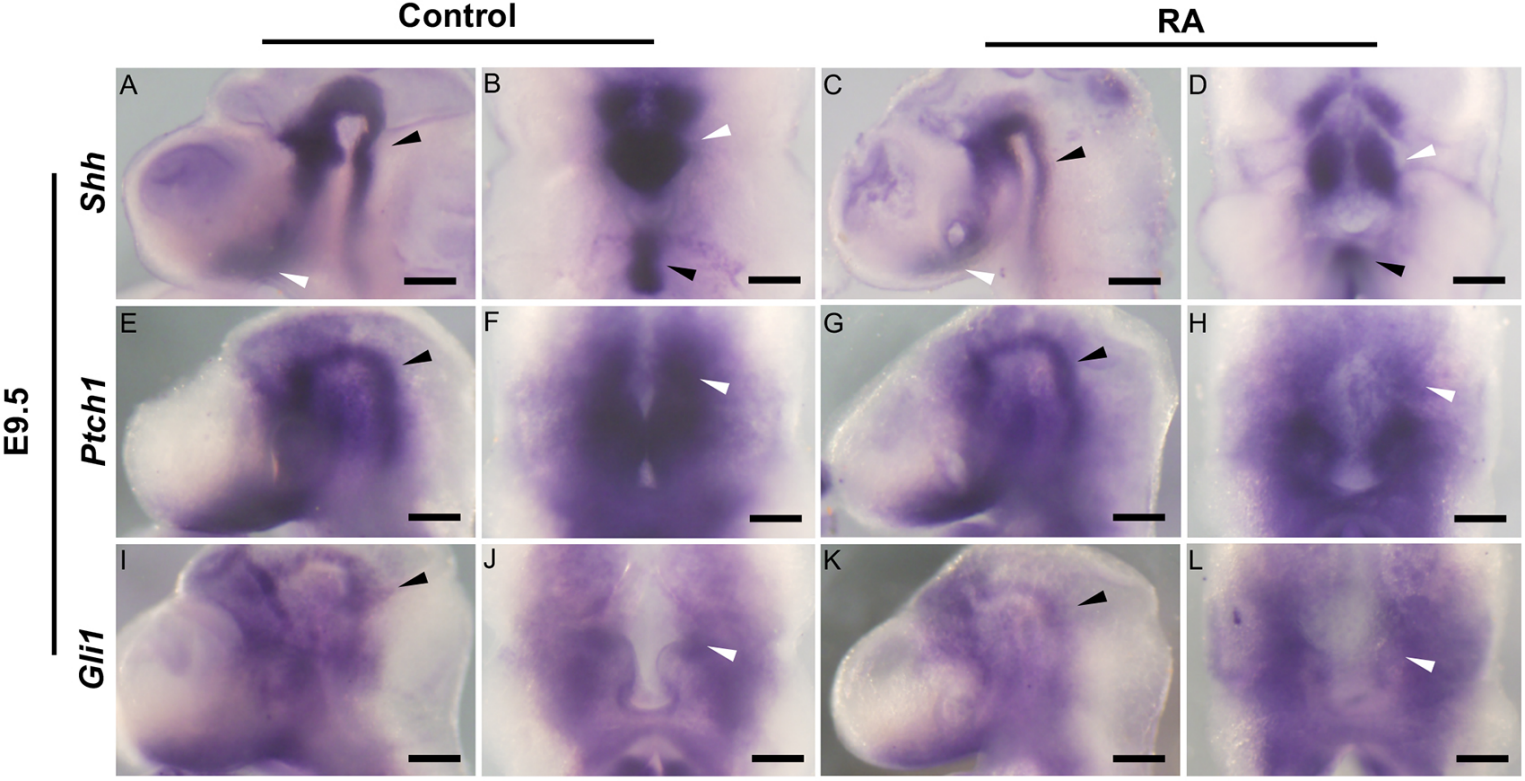
**Expression of SHH signaling in relation to facial development in control and RA-treated embryos.** Lateral views (A,C,E,G,I,K) and ventral views (B,D,F,H,J,L) of whole-mount *in situ* hybridization for *Shh* (A-D), *Ptch1* (E-H) and *Gli1* (I-L) in E9.5 control (A,B,E,F,I,J) and RA-treated (C,D,G,H,K,L) embryos. (A-D) *Shh* expression in the floor plate of the neural tube (black arrowheads) and the ventral forebrain neuroepithelium (white arrowheads) is reduced in RA-treated embryos. *Ptch1* (E-H) and *Gli1* (I-L) expression in the ventral central nervous system (black arrowheads) and the mesenchyme surrounding the notochord adjacent to the *Shh* expression domain (white arrowheads) is slightly lower in RA-treated embryos. Scale bars: 100 µm.

To understand the potential molecular mechanism underlying RA-induced defects in CNCC development during the pathogenesis of CP, we performed comparative RNAseq analyses of the E11.5 maxillary complex, including the medial nasal process (giving rise to the primary palate), lateral nasal process, and maxillary process (giving rise to the secondary palate) (Bush and Jiang, 2012). Comparison of control and RA-treated embryos revealed significant differences in the expression levels of several genes, including *Shh* and single-minded homolog 2 (*Sim2*) (Table S1). *Shh* plays critical roles in craniofacial development by regulating NCC survival, proliferation and patterning (Ahlgren and Bronner-Fraser, 1999; Jeong et al., 2004). Therefore, we examined the spatiotemporal expression patterns of *Shh* and its transcriptional targets *Ptch1* (Goodrich et al., 1997) and *Gli1* (Lee et al., 1997) in E9.5-E10.5 embryos, in association with exposure to RA at E8.5. At E9.5, *Shh* is expressed in the notochord and floor plate of the neural tube (Fig. 3A,B, black arrowhead) extending from the prechordal mesoderm and ventral forebrain caudally (Fig. 3A,B, white arrowhead). In E9.5 RA-treated embryos, *Shh* expression was visibly reduced (Fig. 3C,D, arrowheads). *Ptch1* and *Gli1* expression levels in the ventral central nervous system and the mesenchyme surrounding the notochord (Bai et al., 2002) were also lower in E9.5 RA-treated embryos compared to controls; however, the downregulation seemed to be subtle compared to that of *Shh* expression (Fig. 3E-L). These results suggest that excess RA signaling at E8.5 leads to reduced *Shh* expression in the ventral forebrain region adjacent to the maxillary prominence, which is known to impact the survival of CNCCs, and results in craniofacial anomalies such as CP.

### RA-induced cleft palate and CNCC apoptosis are partially rescued by exogenous SAG (*Shh* agonist) *in utero*

**Fig. 4.**
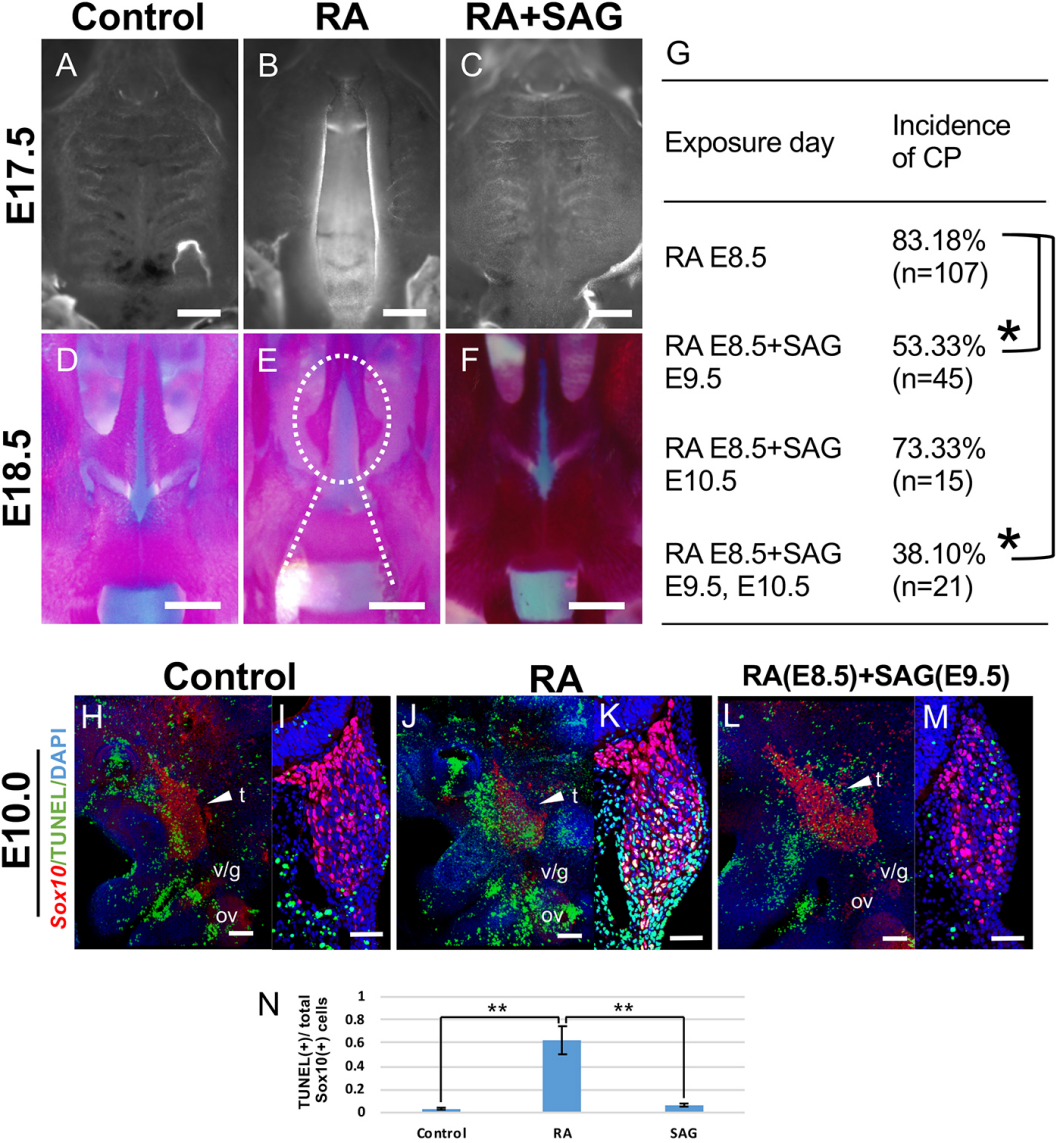
**Exogenous SAG (Shh agonist) partially rescues RA-induced cleft palate (CP) and CNCC apoptosis *in utero*.** (A-C) Ventral views of DAPI-staining of palates in E17.5 control embryos (A), RA-treated embryos (B) and RA-treated embryos with the addition of SAG (RA+SAG) by oral gavage (C). (D-F) Alizarin Red (bone)- and Alcian Blue (cartilage)-stained skeletal preparations of control (D), RA-treated (E) and RA+SAG-treated (F) embryos at E18.5. The failure of maxillary process growth toward the midline (oval dashed line in E) and the presphenoid bone exposure (dashed lines in E) due to CP in RA-treated embryos are rescued by SAG administration (F). (G) The incidence of CP with SAG (12.5 mg/kg body weight) exposure at E9.5 and/or E10.5 after all-trans RA (25 mg/kg body weight) treatment at E8.5. Fisher's exact test was used for statistical analysis. *P<0.01. *n*, number of embryos analyzed; %, percentage relative to the number of embryos analyzed. (H-N) Increased apoptosis in *Sox10*-positive (red) trigeminal ganglia (arrowheads) at E9.5 after RA treatment is rescued by SAG administration. TUNEL (green) detection of whole-mount in profile views (H,J,L) and transverse sections (I,K,M) at the level of the trigeminal ganglia of control (H,I), RA-treated (J,K) and SAG-treated (L,M) E9.5 embryos. Quantification of apoptosis (TUNEL+, N) in *Sox10*-positive CNCCs from control, RA-treated and SAG-treated embryos in the trigeminal ganglia regions. *t*-test **P<0.01, *n*=5. Data are mean±s.e.m. t, trigeminal ganglion; v/g, vestibulo-cochlear/geniculate ganglia; ov, otic vesicle. Scale bars: 500 µm (A-F); 100 µm (H, J and L); 50 µm (I, K and M).

To confirm whether altered Shh signaling underlies the etiology of CP in association with excess RA signaling, we supplemented RA-treated embryos with the Smo activator SAG (Smo agonist). Interestingly, administration of SAG after RA treatment resulted in a reduced frequency of CP at E18.5 (Fig. 4C). Growth of individual maxillary processes towards the midline as well as the fusion of the bilateral palatal bones were indistinguishable in RA-treated embryos and controls (Fig. 4A,D and F). Notably, the frequency of CP depended on the timing of SAG administration (Fig. 4G). In RA-treated embryos, CNCC apoptosis was clearly evident at E9.5. Therefore, we predicted that rescue of CP would require SAG at or around E9.5. We administered vehicle or SAG (12.5 mg/kg body weight) to RA-treated (E8.5) pregnant mice at E9.5 and/or E10.5 by oral gavage (Frank-Kamenetsky et al., 2002). The incidence of CP was significantly reduced by the administration of SAG at E9.5 and E9.5-E10.5 (*P*<0.01, Fig. 4G). Administering SAG at E10.5 also tended to reduce the incidence of CP; however, there was no significant difference compared with the RA-treated group (Fig. 4G). Furthermore, we analyzed the effect of SAG treatment on CNCC survival. TUNEL staining of whole embryos and of transverse sections revealed a significant increase in cell death in the maxillary region in RA-treated embryos at E9.5 (Fig. 4J,K). We found that the addition of exogenous SAG after RA treatment resulted in a considerable reduction in CNCC apoptosis (Fig. 4L,M). Statistical analyses revealed that exogenous SAG was able to rescue the RA-induced decrease in CNCC survival in the maxillary process, such that it was comparable to controls (Fig. 4N). Taken together, these results demonstrate that disruption of Shh signaling at around E9.5-E10.5 in association with excess RA signaling underlies the pathogenesis of CP.

## DISCUSSION

In this study, we obtained molecular and cellular evidence illustrating how exposure to RA at an early gestational stage (from E8.5 to E10.5) can result in CP in mice. More specifically, we determined that E8.5 is the most sensitive time point at which RA treatment during embryogenesis results in CP together with defects in the NCC-derived craniofacial skeleton. Furthermore, we showed that excess RA signaling alters Shh signaling in craniofacial tissues, which induces apoptosis of CNCCs that would normally contribute to the maxillary process. Thus, RA signaling plays a critical role in regulating CNCC survival during palatogenesis and craniofacial skeleton development via Shh signaling.

### Stage-dependent effects on RA-induced cleft palate

Since excess RA was shown to be a teratogen that can cause CP in conjunction with other congenital malformations in rodents (Kochhar and Johnson, 1965), numerous studies have attempted to elucidate the stage-specific effects of excess RA signaling in the pathogenesis of CP. Excess RA exposure at E9 was shown to induce high levels of apoptosis in the first branchial arch, which reduced the volume of the secondary palate tissues (Sulik et al., 1987). In comparison, when mouse embryos were treated with RA at E10, the treatment resulted in decreased mesenchymal proliferation and a failure of the small palatal shelves to contact each other (Abbott and Birnbaum, 1990). Meanwhile, later treatments (E11.5-E12.5) have been shown to prevent palatal shelf elevation by affecting the extracellular matrix composition and/or tongue descension by disturbing tongue muscle development (Degitz et al., 1998; Okano et al., 2008, 2007). We show herein that the incidence of CP was highest in offspring of pregnant mice treated at E8.5 (83.18%), or repeatedly at E8.5 plus a later time point (E8.5&E9.5, 95.56%; E8.5&E10.5, 97.73%; E8.5&E9.5&E10.5, 100%), whereas treatment only at these later time points (E9.5&E10.5) resulted in a less frequent incidence of CP (55%; Fig. 1E). It is also important to mention the possibility that some of the embryos judged negative for CP still have submucous CP. These results strongly suggest that excess RA signaling at E8.5 is a critical factor in the pathogenesis of CP. It is well known that, at around E8.5, the formation of CNCCs and the onset of their migration takes place, both of which are critical steps in craniofacial development (Trainor, 2005). Therefore, we hypothesized that excess RA signaling at E8.5 could negatively impact CNCC development.

Previous *in vitro* and *in vivo* studies have shown that proper RA signaling is essential for normal NCC development. Targeted inactivation of *Rdh10* or *Aldh1a2*, the enzymes responsible for converting vitamin A to RA during embryogenesis, results in agenesis of the posterior branchial arches (BA3-BA6) in E9.5 mouse embryos (Niederreither et al., 2003; Sandell et al., 2007). Our present study shows that excess RA signaling results in reduced condensation or contribution of CNCCs to the cranial ganglia. Moreover, in RA-treated embryos, we detected a significant elevation of apoptosis in Sox10-labeled CNCCs in the facial processes and pharyngeal arch mesenchyme. These data highlight the fact that defective palatogenesis in embryos treated with RA at E8.5 *in utero* is associated with dysregulated CNCC development. One possible explanation for the higher rates of defective palatogenesis in embryos treated with RA at E8.5 and subsequently at E9.5 and/or E10.5 is the synergistic or combined effects of the sequential treatments.

### Proper RA signaling is required for SHH signaling in the craniofacial region

**Fig. 5.**
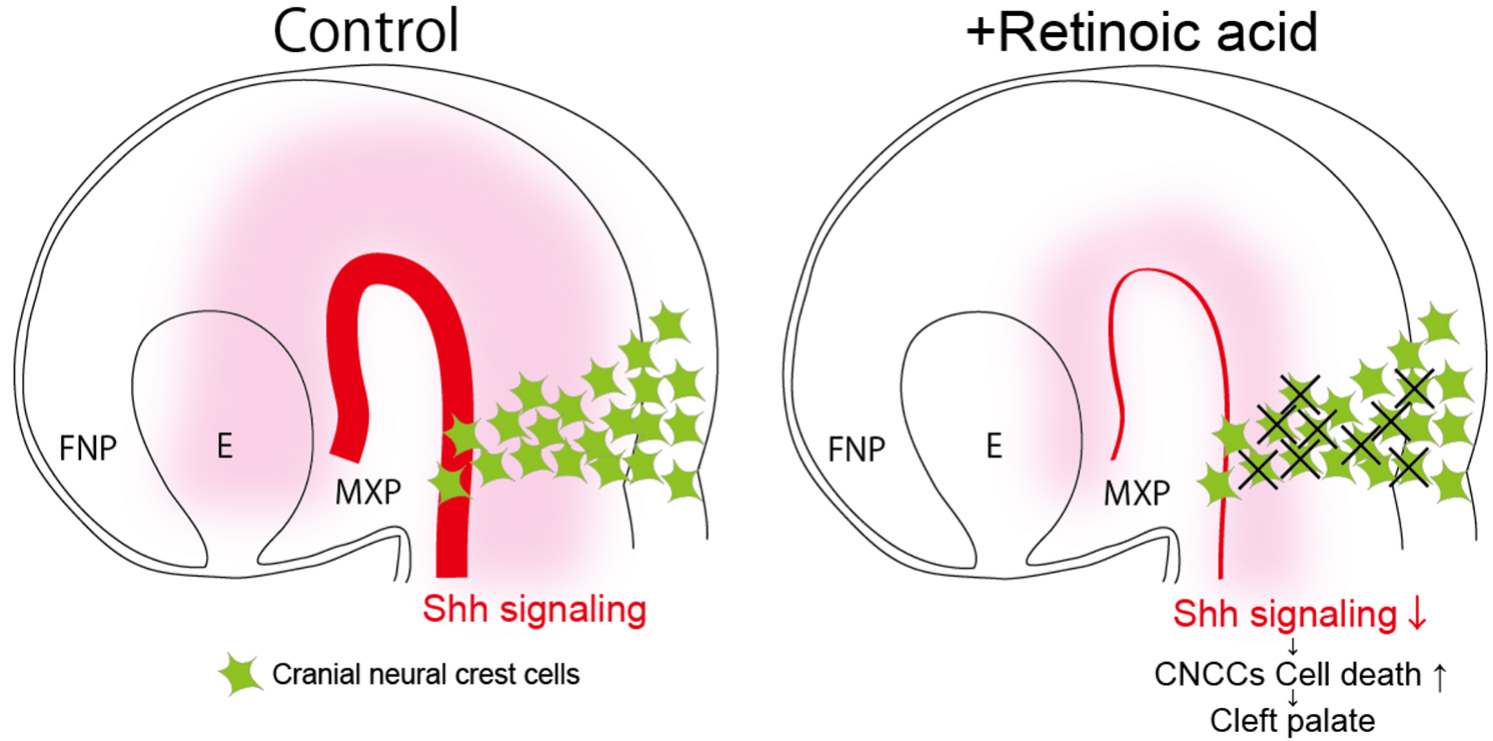
**Excessive RA signaling reduces Shh signaling, which results in elevated cell death of CNCCs and cleft palate.** Proper Shh signaling is essential for the survival of CNCCs, which contribute to secondary palate development (left side). Excessive RA signaling reduces Shh signaling and elevates the cell death of CNCCs, which results in cleft palate (right side). FNP, frontonasal process; MXP, maxillary process; E, eye.

Previous studies have shown that *Shh* and RA signals interact and influence many developmental processes. In particular, in the craniofacial region, local synthesis of RA in the rostral region of the chick embryo head enables patterned outgrowth of the forebrain and frontonasal process via maintenance of Shh signaling (Schneider et al., 2001). The present study revealed altered expression of *Shh* and its downstream genes *Ptch1* and *Gli1* in the ventral region of the neural tube in E9.5 RA-treated embryos. Altered Shh signaling is well known to be associated with a variety of craniofacial defects, including facial clefts (Kurosaka, 2015; Kurosaka et al., 2014; Rice et al., 2004). Additionally, it has been reported that proper Shh signaling is essential for the survival of CNCCs (Ahlgren and Bronner-Fraser, 1999; Moore-Scott and Manley, 2005). From these results, it can be concluded that at least one reason for elevated CNCC death in RA-treated embryos is the reduction in Shh signaling, which likely contributes to the etiology of CP (Fig. 5). Additionally, crosstalk between RA and Shh signaling in the developing maxillary region could occur similarly in certain different embryonic stages, according to previous findings and our RNAseq data from E11.5 (Helms et al., 1997).

To specifically test this mechanism, we supplemented RA-treated embryos with an Shh signaling agonist (SAG), which directly binds the Smo receptor and activates the pathway. Previous studies using *Shh^+/−^* or *Shh*^−/−^ embryos showed that the midline defects are at least partly rescued by SAG treatment (Frank-Kamenetsky et al., 2002). It has also been shown that SAG activates the Shh signaling pathway during neurogenesis and augments neuronal survival (Bragina et al., 2010). Importantly, we showed here that repeated exogenous SAG (12.5 mg/kg body weight) administration at E9.5 and E10.5 significantly reduced the incidence of CP in association with RA exposure. Interestingly, administering SAG only at E10.5 did not elicit a significant difference in the prevalence of CP, which indicates that there is a precise temporal requirement for Shh signaling during embryogenesis for normal palate development. It is also possible that better rescue efficacies might be obtained if SAG administration were performed at an even earlier stage. These results again emphasize the significance of reduced Shh signaling and subsequent increased CNCC death in the pathogenesis of CP. However, the occurrence of CPs was not completely prevented by SAG supplementation. One possible explanation for the partial rescue is that RA may also regulate other genes associated with palatogenesis. For example, *Sim2* is downregulated in RA-treated embryos according to our RNAseq analyses. Furthermore, mice homozygous for a deletion in *Sim2* exhibit a cleft of the secondary palate and other craniofacial malformations (Shamblott et al., 2002). Since RA signaling is well known to affect many signaling pathways, further investigation will be required in order to reveal whether other molecular causes or mechanisms result in CP in association with an excess of RA signaling.

We have also detected several different phenotypes in RA-treated embryos, such as cranial muscle defects and syngnathia to different degrees (data not shown). Proper patterning of cranial muscle and jaws is regulated by various signaling pathways (Inman et al., 2013). Since RA signaling is known to crosstalk with various molecular networks, further investigations will be required to reveal other molecular etiologies underlying these conditions.

## MATERIALS AND METHODS

### Animals, and RA and SAG administration

Pregnant female Institute of Cancer Research (ICR) mice (CLEA, Japan) were administered all-trans RA (25 mg/kg body weight) (Sigma-Aldrich) by oral gavage. All of the mice were housed with a 12 h dark-light cycle in which the light phase started from 8 am. RA (25 mg/ml in dimethylsulphoxide) was diluted 1/10 in corn oil just before use. Control animals were given the equivalent volume of the carrier. Oral gavage was performed once per day at different gestation stages (from E8.5 to E10.5) (Fig. 1E). Embryonic stage E0.5 was defined on the morning of vaginal plug confirmation. The approximate somite number in ICR mice at E8.5 was seven pairs.

An Shh signaling chemical agonist, SAG (Enzo), was administered once per day by oral gavage at E9.0, E9.5 and/or E10.5 after RA treatment at E8.5. SAG solution was prepared as a fine suspension in 0.5% methylcellulose/0.2% Tween 80 at 1.5 mg/ml and given to pregnant mice at 100 µl per 10 g body weight (Frank-Kamenetsky et al., 2002).

The evaluation of palatal shelf development was performed after E16.0, and only the complete cleft of the secondary palate detected by visual observation was defined as CP. All animal experiments were performed in accordance with the guidelines of the Animal Care and Use Committee of the Osaka University Graduate School of Dentistry, Osaka, Japan. The committee on the ethics of animal experiments of the same university approved the study protocol (permit number: 26-028-0).

### Bone and cartilage staining

The embryos were skinned and fixed in 100% ethanol overnight and then stained for 24 h in Alcian Blue (150 µg/ml in 20 ml of glacial acetic acid and 80 ml of 95% ethanol). After washing in 100% ethanol, soft tissues were dissolved in 2% KOH overnight and stained with Alizarin Red (50 µg/ml in 1% KOH) overnight.

### Whole-mount *in situ* hybridization

Whole-mount *in situ* hybridization was performed as described (Wilkinson, 1992) with minor modifications using digoxigenin (DIG)-UTP (Roche)-labeled antisense RNA probes corresponding to the sequences of *Sox10*, *Tfap2a*, *Shh*, *Ptch1* or *Gli1* (sequences used were previously reported in the Allen Brain Atlas: http://portal.brain-map.org/). For all *in situ* hybridization analyses, a minimum of three embryos of each sample were examined per probe.

### DAPI staining and analysis of apoptosis and cell proliferation

For whole-mount DAPI staining to visualize palate tissues (Sandell et al., 2012), E15.5 and E17.5 embryos were fixed in 4% PFA and incubated overnight in 1:1000 DAPI dilution (Dojindo).

Analyses of apoptotic cells were performed using an *in situ* cell death detection kit (www.Roche.com) following the manufacturer's instructions. For analyses of proliferation, samples were incubated with a mouse anti-pHH3 antibody (1:200, Millipore) at 4°C overnight followed by secondary Alexa-Fluor-488 donkey anti-mouse IgG (1:200, Invitrogen) for 6 h at room temperature for sections and overnight at 4°C for whole embryos. To label NCCs, sections were counterstained with a goat anti-Sox10 antibody (5 µg/ml, R&D Systems) at 4°C overnight, followed by secondary antibody (Alexa-Fluor-546 donkey anti-goat IgG, 1:200, Molecular Probes). Cells in at least five adjacent sections were counted in each assay. Statistical significance was assessed using a two-tailed Student's *t*-test.

### RNAseq analysis

Medial nasal, lateral nasal and maxillary processes were separated from E11.5 control (*n=*3) and RA-treated (*n=*3) embryos. RNAseq analysis was performed as previously described (Kurosaka et al., 2017).

## Supplementary Material

Supplementary information
