# Supplementary material for: Perturbed development of cranial neural crest cells in association with reduced sonic hedgehog signaling underlies the pathogenesis of retinoic-acid-induced cleft palate
Source: Dis Model Mech. 2019 Oct 4;12(10):dmm040279. doi: 10.1242/dmm.040279 (PMC6826016; doi:10.1242/dmm.040279)
Supplement: Supplementary information [file dmm-12-040279-s1.pdf]

down – regulated gene	Log2FC (Log2FC < -1 or Log2FC > 1)
Sim2	-2.39
Ugt2a1	-1.95
Cyp26c1	-1.54
Sult1e1	-1.12
mt-Tl1	-1.09
Shh	-1.06
Gm129	1.02
Gm14506	1.26

Table S1. Differentially expressed transcripts in the medial nasal process, the lateral nasal process, and the maxillary process of RA-treated embryos at E11.5. Transcripts were selected by identifying those having a fold change Log2FC < -1 or Log2FC > 1 in RA-treated versus control embryos, as described in the Methods section.
